# Small RNAs and the regulation of cis-natural antisense transcripts in Arabidopsis

**DOI:** 10.1186/1471-2199-9-6

**Published:** 2008-01-14

**Authors:** Hailing Jin, Vladimir Vacic, Thomas Girke, Stefano Lonardi, Jian-Kang Zhu

**Affiliations:** 1 Departments of Plant Pathology & Microbiology, Center for Plant Cell Biology and Institute for Integrative Genome Biology, University of California, Riverside, CA 92521, USA; 2 Computer Science and Engineering, University of California, Riverside, CA 92521, USA; 3 Botany and Plant Sciences, Center for Plant Cell Biology and Institute for Integrative Genome Biology, University of California, Riverside, CA 92521, USA; 4 Computer Science and Engineering, Center for Plant Cell Biology and Institute for Integrative Genome Biology, University of California, Riverside, CA 92521, USA

## Abstract

**Background:**

In spite of large intergenic spaces in plant and animal genomes, 7% to 30% of genes in the genomes encode overlapping cis-natural antisense transcripts (cis-NATs). The widespread occurrence of cis-NATs suggests an evolutionary advantage for this type of genomic arrangement. Experimental evidence for the regulation of two cis-NAT gene pairs by natural antisense transcripts-generated small interfering RNAs (nat-siRNAs) via the RNA interference (RNAi) pathway has been reported in Arabidopsis. However, the extent of siRNA-mediated regulation of cis-NAT genes is still unclear in any genome.

**Results:**

The hallmarks of RNAi regulation of NATs are 1) inverse regulation of two genes in a cis-NAT pair by environmental and developmental cues and 2) generation of siRNAs by cis-NAT genes. We examined Arabidopsis transcript profiling data from public microarray databases to identify cis-NAT pairs whose sense and antisense transcripts show opposite expression changes. A subset of the cis-NAT genes displayed negatively correlated expression profiles as well as inverse differential expression changes under at least one of the examined developmental stages or treatment conditions. By searching the *Arabidopsis *Small RNA Project (ASRP) and Massively Parallel Signature Sequencing (MPSS) small RNA databases as well as our stress-treated small RNA dataset, we found small RNAs that matched at least one gene in 646 pairs out of 1008 (64%) protein-coding cis-NAT pairs, which suggests that siRNAs may regulate the expression of many cis-NAT genes. 209 putative siRNAs have the potential to target more than one gene and half of these small RNAs could target multiple members of a gene family. Furthermore, the majority of the putative siRNAs within the overlapping regions tend to target only one transcript of a given NAT pair, which is consistent with our previous finding on salt- and bacteria-induced nat-siRNAs. In addition, we found that genes encoding plastid- or mitochondrion-targeted proteins are over-represented in the Arabidopsis cis-NATs and that 19% of sense and antisense partner genes of cis-NATs share at least one common Gene Ontology term, which suggests that they encode proteins with possible functional connection.

**Conclusion:**

The negatively correlated expression patterns of sense and antisense genes as well as the presence of siRNAs in many of the cis-NATs suggest that siRNA regulation of cis-NATs via the RNAi pathway is an important gene regulatory mechanism for at least a subgroup of cis-NATs in Arabidopsis.

## Background

Natural antisense transcripts (NATs) are a class of endogenous coding or non-coding RNAs that have sequence complementarity to other RNAs in the cell. *Cis*-NATs are transcribed from the opposite strands of the same genomic locus, in which case the sequence complementarity between two transcripts is directly related to the overlap in locations of the corresponding genes on the sense and antisense strands. In contrast, *trans-*NATs are transcribed from different genomic loci. Cis-NATs usually have a long perfect complementary overlap between the sense and antisense transcripts, whereas the trans-NATs often have short and imperfect complementarity. Recent genome-wide analyses revealed a surprisingly widespread existence of NATs in eukaryotic genomes [1-4], which suggests that NATs may have evolved as a common regulatory mechanism for gene expression. Approximately 22–26% of human genes [5-7], 14.9–29% of mouse genes [5,8-10], 15.4–16.8% of Drosophila genes [5,11], 8.9% of Arabidopsis genes [12,13] and 7% of rice genes are overlapping [1,14] and form *cis-*NATs. Recently, many new cis-NATs were identified from the Massively Parallel Signature Sequencing (MPSS) datasets of human and mouse NATs [15]. The authors suggested that alternative polyadenylation and retroposition may account for the origin of a significant number of functional sense-antisense pairs in mammalian genomes. In spite of large intergenic spaces, many genes are still overlapping, such a genomic arrangement must be functionally beneficial. NATs are involved in diverse physiological processes, including pathophysiological processes in human diseases [16]. Despite the widespread of NATs in eukaryotic genomes, the mechanisms of their regulation are still largely unknown.

NATs have been implicated in various aspects of expression regulation of eukaryotic genes, including transcriptional interference [17], RNA masking-induced alternative splicing [18], X chromosome inactivation [19-21], genomic imprinting [22-24], RNA editing [25,26] and small RNA-induced gene silencing [16,27-29]. Recent analysis showed that 47% of the human imprinted genes in the IGC database are arranged as NATs [5].

In small RNA-induced gene silencing pathways, double-stranded RNAs (dsRNAs) can be processed by RNase III enzymes known as Dicers or Dicer-like proteins into small interfering RNAs (siRNAs) [30,31]. The siRNAs are then incorporated into the argonaute-containing RNA-induced silencing complex (RISC), to guide the cleavage of complementary mRNAs, or argonaute-containing RNA-induced transcriptional silencing complex (RITS), to guide chromatin modifications [30,31]. Co-expression of overlapping sense/antisense transcripts could potentially form dsRNAs, which may be recognized by Dicer or Dicer-like proteins and processed into siRNAs. Recently, we found two pairs of cis-NATs in Arabidopsis that generate siRNAs from the sense strand (termed natural antisense transcript-siRNAs [nat-siRNAs]), which then target the antisense strand for cleavage [28]. In these two cases, the sense transcript is induced by abiotic or biotic stress and the resulting nat-siRNAs cause the silencing of the constitutively expressed antisense transcript. These results suggest that the inverse changes in gene expression within a NAT pair (i.e. induction of one strand and silencing of the other), may be a diagnostic feature of RNAi regulation of NATs. A genome-wide analysis of human cis-NATs has indicated that the genes within a NAT pair tend to be inversely expressed and/or co-expressed more frequently than expected by chance [32], although whether any NATs are regulated by RNAi in humans is unclear.

The proportion of inversely expressed cis-NATs in the Arabidopsis genome is unknown, and the extent of small RNA-mediated gene regulation within the NAT genes is completely unknown in any organism. Recent studies employing high throughput sequencing of small RNAs have identified endogenous small RNAs matching many protein coding genes in Arabidopsis [33-35]. We were interested in determining whether cis-NAT genes are equally prone to small RNA regulation, or are deficient or enriched for small RNA matches. The identification of NATs with inverse expression patterns and small RNAs that match the NATs would provide an indication of RNAi regulation of the NATs.

To determine the extent of regulation of cis-NATs by RNAi, we conducted a genome-wide analysis of Arabidopsis cis-NAT gene expression and identified a large number of siRNAs that match these cis-NATs. We found a subgroup of cis-NATs with negative co-regulation between their sense and antisense partners that are enriched for small RNA matches. In general, cis-NAT protein-coding genes have a slightly higher frequency of small RNA matches than non-NAT protein-coding genes. We conclude that many cis-NAT genes may be regulated by siRNAs in response to various environmental or developmental cues.

## Results

### Identification of Arabidopsis cis-NATs with inverse expression changes in response to various environmental and developmental conditions

**Figure 1:**
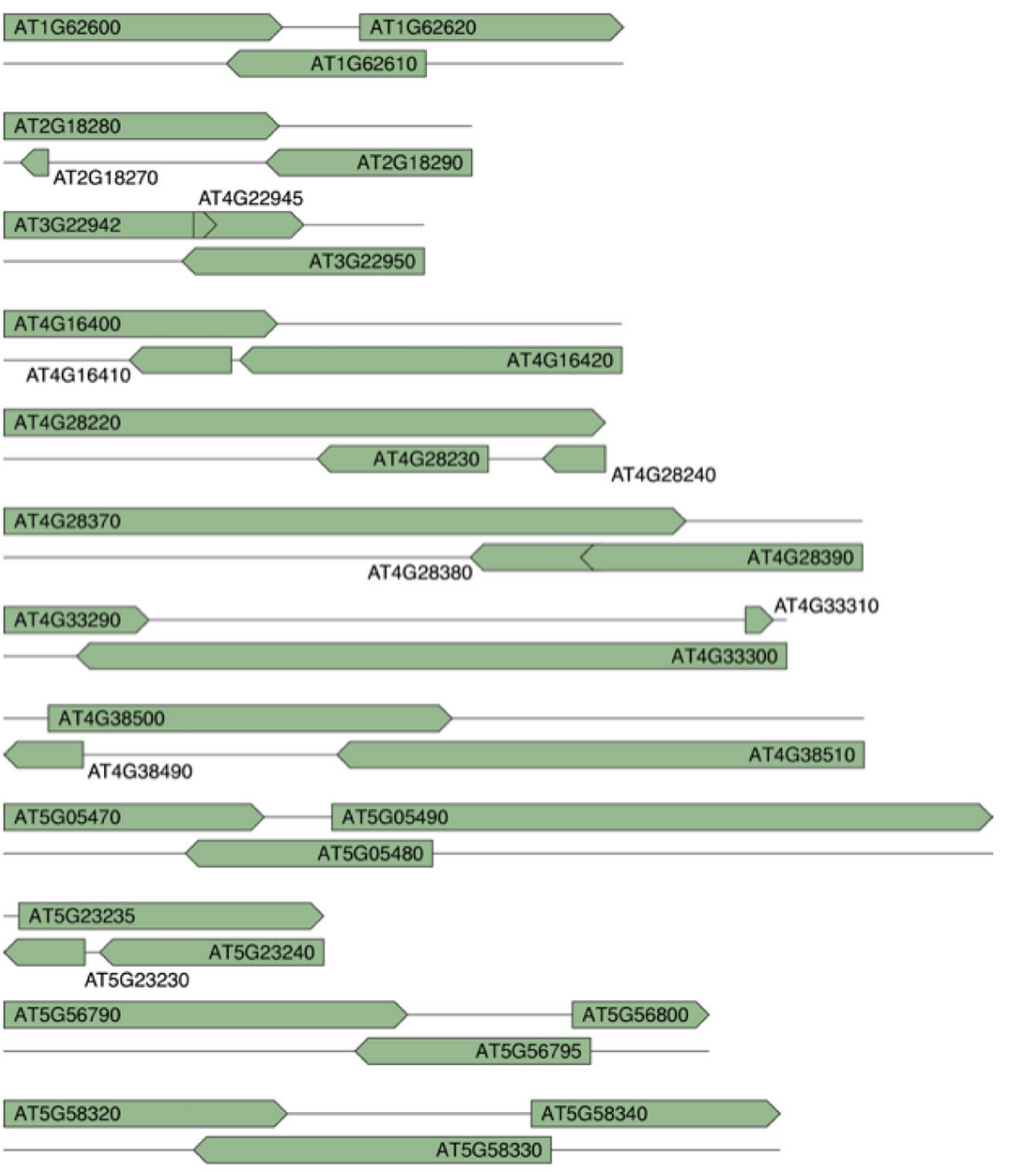
Twelve genes that overlap with two antisense transcripts. The point ends represent the 3' end of the gene.

Co-expression of sense and antisense transcripts within a NAT pair could potentially form dsRNAs, which could be processed into small RNAs and cause silencing of the antisense transcript. We hypothesized that the expression of many NAT genes may be induced under specific environmental or developmental conditions to avoid the cost of constant silencing caused by sense-antisense transcript pairing. To test this hypothesis, we identified all the cis-NATs in the Arabidopsis genome and conducted computational analysis of large-scale microarray expression data from public databases. We searched The Arabidopsis Information Resource (TAIR, 6.0 annotation) and identified 2095 NAT pairs with a minimum overlap of 20 base pairs (bp), including both cis- and trans-NATs. When loci of several splice variants overlapped, gene models with the longest overlapping region were chosen. Of the 2095 pairs, 1057 pairs are cis-NATs, that is, the two genes within the NAT pair are located on the opposite strands of the same genomic locus. These cis-NATs include 1008 pairs with overlap between protein-coding genes, 32 with overlap between protein-coding and t/sn/snoRNA genes, 2 involving trans-acting siRNA (ta-siRNA) primary transcripts and 15 pairs involving transposon genes (Table 1 and Additional file 1). Most of the cis-NATs (941 pairs) are arranged in convergent orientation, with the 3' ends overlapping. The overlap between sense and antisense transcripts in 204 pairs involves introns. Interestingly, we found 12 genes overlapping with two distinct genes on the opposite strands (Figure 1). In this study, we mainly focused on the protein-coding cis-NATs.

**Table 1:** List of Arabidopsis NATs. (Based on TAIR 6.0 dataset "AGI Genes", which contains gene sequences inclusive of introns and UTRs.)

**Arabidopsis NATs gene arrangement**	**Convergent**	**Divergent**	**Enclosing**	**Total (pairs)**	**Overlap length (mean ± stdev)**
All NATs (pairs)	1823	108	164	2095	402 ± 524

cis-NATs (pairs)	941	41	75	1057	472 ± 401

**PC/PC**	**926**	**35**	**47**	**1008**	**485 ± 387**
PC/tRNA	3	0	22	25	69 ± 11
PC/ta-siRNA	1	0	1	2	143 ± 158
PC/snRNA	3	2	1	6	241 ± 248
PC/snoRNA	0	0	1	1	121 ± 0
PC/transp	7	2	2	11	584 ± 1178
transp/tRNA	0	0	1	1	71 ± 0
transp/transp	1	2	0	3	81 ± 31

PC: protein coding gene

To determine the extent of small RNA-mediated gene silencing in regulation of cis-NAT genes, we performed correlation and differential expression analyses of 1310 Affymetrix ATH1 genome arrays derived from 41 different experiment series from the AtGenExpress [36] and GEO sites [37] (Additional file 2). We performed statistical analysis of differentially expressed genes only on the experiment sets with two or more replicates. The selected replicated experiment sets included mutant and tissue comparisons, as well as sample sets that were treated with different abiotic stresses, biotic stresses, chemicals and hormones (Additional file 2). All NAT pairs represented on the ATH1 chip by ambiguous probe sets (*e.g*. probe sets targeting several genes) were excluded from the subsequent steps of the analysis. The remaining 666 NAT pairs with unambiguous probe sets were used for correlation and differentially expressed gene (DEG) analyses. To identify global negative co-regulation effects between NAT pair members, we calculated the Pearson and Spearman correlation coefficients for the expression profiles of sense and antisense partners of each gene pair. We used the averaged intensity values between replicates of the total dataset (Additional file 3) for this analysis. The obtained correlation coefficients provide a measure of the similarity of expression profiles. Correlation values close to +1 indicate positive correlation, values close to 0 imply no correlation and values close to -1 indicate negative correlation. We identified 148 NAT pairs with a Pearson correlation coefficient ≤ -0.2 and 201 pairs with a Spearman correlation coefficient ≤ -0.2; with a correlation cutoff of ≤ -0.4, we identified 71 and 100 pairs, respectively. Interestingly, about 85% of the identified negatively co-regulated gene pairs were also identified by the differential expression analysis (low stringency type) described later. When the averaged gene expression levels across all experiments are divided into the three expression classes high, medium and low, 52% of the negatively correlated genes from the Pearson method are expressed at high, 35% at intermediate and 13% at low levels (see Supplement Additional file 3). For the 201 pairs from the Spearman method it is 47%, 38% and 15%, respectively. These results indicate that the many negatively correlated NAT pair members are expressed at intermediate or high levels.

**Figure 2:**
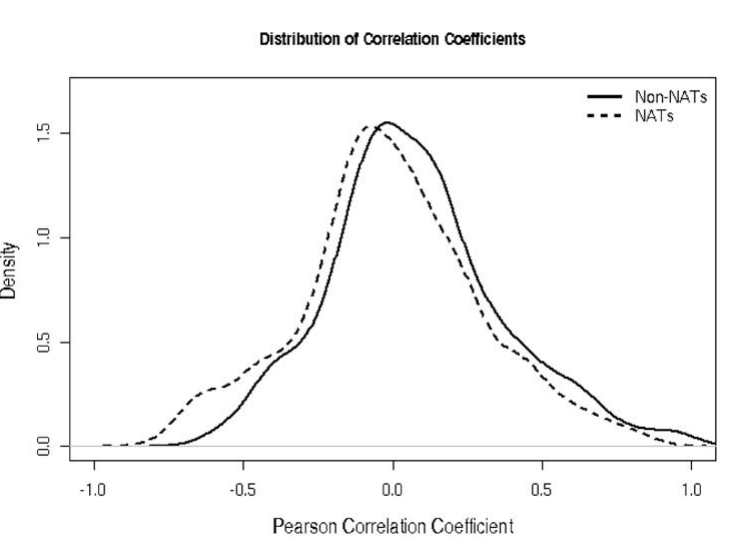
Pearson correlation coefficients for the cis-NAT pairs and non-overlapping gene pairs plotted to form a density plot. The corresponding means, standard deviations and p-values are in Table 2.

Figure 2 shows that the distribution of the correlation coefficients of the cis-NAT dataset is slightly shifted toward lower coefficients as compared with the corresponding data set for all non-overlapping gene pairs in the Arabidopsis genome. The very low p-value of a two-sample t-test (Table 2) indicates that this difference is statistically significant, whereas the difference for randomized gene pairs (500 iterations) is less pronounced. We also observed slight positive correlations between neighboring genes, which may be explained by common chromatin configurations, transcriptional hot spots or other factors.

**Table 2:** Correlation Analysis of NAT Pairs

	**mean**			**p-value (NATs vs.)**	
		
	**Random**	**NATs**	**Neighbor**	**Neighbor**	**Random**
**Pearson**	0.022 ± 0.011	-0.006 ± 0.304	0.082 ± 0.298	7.55E-13	0.163
**Spearman**	0.022 ± 0.013	0.001 ± 0.352	0.082 ± 0.332	1.20E-08	0.351

The mean Pearson and Spearman correlation coefficients with their standard deviations for randomized (Random, mean of 500 iterations), NAT and all non-overlapping gene pairs (Neighbor). A two-sample t-test was used to calculate p-values for differences in distribution of coefficients between the cis-NAT dataset and the other two datasets.

We also attempted to identify specific inverse expression changes between NAT sense and antisense transcripts by DEG analysis using as cutoff an adjusted p-value of the Limma package of ≤ 0.01 and a fold change of at least 2 [38]. This stringent strategy identified 155 pairs with at least one inverse expression change at specific comparisons (*e.g*. stress level and duration; Additional file 3). Of these 155 pairs, 54 had a negative (≤ -0.2) and 25 a positive (≥ 0.2) Pearson correlation coefficient. Because the silencing of an antisense target may occur at later time points or stages, we also identified slow expression responses with inverse fold changes between sense and antisense transcripts anywhere (*i.e*. not necessarily comparing the same stress level or duration) within a given experiment set using the same cutoff parameters (low stringency type). This less stringent approach identified 593 pairs with at least one inverse expression change anywhere within an experiment set (Additional file 3). Of these 593 pairs, 139 had a negative (≤ -0.2) and 123 a positive (≥ 0.2) Pearson correlation coefficient. Thus, many NAT pairs display inverse expression changes in response to specific environmental or developmental cues. However, as shown in Table 3, the number of observed NAT gene pairs with opposing expression changes is not significantly greater than that obtained from randomized datasets consisting of artificially joined gene pairs. The result indicates that the DEG analysis as applied here cannot be used to reliably identify inversely regulated cis-NATs. Nevertheless, the NAT pairs identified by the DEG analysis as having inverse expression profiles may still be useful for experimental validation as putatively inversely regulated NATs.

**Table 3:** NAT Pairs with Opposite Differential Expression Changes

	**Observed**	**Randomized**	
		
	**N**	**mean**	**p-value**
Comp	155	159.0 ± 9.0	0.703
Exp Set	593	585.3 ± 5.7	0.097

The number (N) of cis-NAT pairs with opposite differential expression changes are given for the stringent (Comp) and the less stringent (Exp Set) approach. The same counts were calculated for randomized gene pairs (1000 iterations). Their mean values and standard deviations (± SD) are provided. The p-values represent the number of times the randomized data generated a value ≥ the observed value (N) divided by 1000.

In summary, the data from both correlation analysis and differential expression study show small differences in opposite expression patterns between cis-NAT, randomized and non-overlapping gene pair samples. Consistent with results of a recent study of expression patterns of NATs [39], these results indicate that negative regulation mechanisms may not be shared by most cis-NAT pairs but are restricted to a subgroup of this gene class that exhibits opposite expression profiles. Alternatively, many cis-NATs might display subtle negative co-regulations only under specific developmental stages or environmental conditions. In the latter case, the associated responses can only be detected by detailed differential gene expression analyses and not by global correlation analyses between pair members. In addition, there may be NAT pairs in which both of the two overlapping genes show an overall positive correlation, but one of the gene members may be strongly induced only under specific conditions. The specific induction of the one member could reach a threshold and result in the down-regulation of its antisense partner by the formation of nat-siRNAs. Finally, we would like to point out that small RNA-mediated gene regulation of NATs occurs only when both transcripts are expressed within the same cell.

### Many NATs can generate siRNAs

Our recent studies suggest that the expression of NATs may be regulated by endogenous siRNAs [28,29]. We searched several publicly available datasets – the *Arabidopsis *Small RNA Project (ASRP) [40] (including recently published datasets [34,41]), MPSS [33,42] and a dataset recently published by Bartel and coworkers (abbreviated as DB) [35] – for small RNAs with 100% sequence match to Arabidopsis cis-NATs. NATs that match transposon, retrotransposon, rRNA, tRNA, snRNA or snoRNA genes were removed prior to this analysis. We identified 3405 unique small RNAs, including 879 and 2115 small RNA sequences from the ASRP and DB datasets, respectively, and 497 unique signatures from the MPSS dataset (Table 4 and Additional file 4), which match a total of 890 NAT genes (Table 5 and Additional file 5). Among them, 828 small RNAs match 165 NAT overlapping regions (Table 4 and Additional file 4).

**Table 4:** Total endogenous small RNAs from ASRP, MPSS, DB and our stress library dataset that match cis-NAT genes in Arabidopsis.

Number of siRNAs that match	Stress	ASRP	MPSS	DB	Unique total
AGI genes	8,862	65,439	31,095	118,825	213,117
PC genes	1,891	37,010	16,657	64,099	115,709
NAT full-length genes	87	879	497	2115	3492
NAT overlapping regions	16	167	76	598	844

**Table 5:** Number of genes with small RNA hits in datasets.

**Genes with small RNA hits**	**Stress**	**ASRP**	**MPSS**	**DB**	**Total (Unique)**
AGI genes	2,596	9,320	8,195	11,516	15,291
PC genes	1,320	6,351	5,083	8,455	12,072
NAT genes	86	382	245	608	914
NAT full-length genes	69	298	193	448	646
NAT overlapping regions	15	65	37	127	180

AGI genes: from TAIR 6.0. PC genes: Protein-coding genes.

Although current ASRP, MPSS and DB small RNA datasets were generated from different developmental stages/tissues (including seedlings, leaves and inflorescence) or from different small RNA biogenesis mutants, very little stress-treated material was included [33-35,40,41,43]. Because many NATs appear to be under the regulation of various abiotic and biotic conditions (Additional file 3), we expected to identify more NAT associated siRNAs from plants treated with various stress conditions. We performed a pilot small RNA profiling experiment on stress-treated plants using 454 sequencing [44,45]. The stress conditions included both abiotic (cold, drought, salt, copper, UV and ABA treatments) and biotic stresses (infection by bacteria *Pseudomonas syringae pv tomato (P.s.t.) strain DC3000 *and *DC3000 *carrying an avirulence gene *avrRpt2*, or fungi *Alternaria brassicicola *and *Botrytis cinerea*). Of 29,556 unique small RNAs with a BLAST hit against the *Arabidopsis *genome (Table 6), we identified 87 new small RNAs that match the NATs but are not present in the ASRP, MPSS or DB datasets. The new small RNAs from the stress libraries match 86 NAT genes, with 16 located in 15 distinct overlapping regions (Table 4, Additional file 4 and Additional file 5). These stress library-specific small RNAs match 24 new NAT genes (Additional file 5). These results, together with the little overlap among these datasets, suggest that the small RNA population identified from these datasets is far from saturation.

**Table 6:** Summary of the numbers of reads from stress-treated small RNA libraries obtained by 454 sequencing

**Small RNA libraries**	**Abio 1**	**Abio 2**	**Abio 3**	**Abio 4**	**Bio 1**	**Bio 2**
Small RNA reads (>= 18)	21203	26246	17824	12465	33289	37599
Unique reads (duplicates removed)	13542	15221	11262	8325	15774	26806
Match genomic DNAs	5431	5882	4514	4198	6236	9613
Match genes + introns + UTR	2917	3183	2444	2102	3933	5914
Match genes - introns + UTR	2814	3063	2357	2038	3873	5667
Matching NAT genes	30	22	19	19	17	24

Abiotic stress-treated libraries

Abio1: Cold treatment, 4°C

Abio2: Draught-treatment and ABA treatment

Abio3: Salt treatment and copper treatment

Abio4: UV treatment and heat treatment

Bio1: *Pseudomonas syringae *DC3000, and DC3000 (avrRpt2) infection

Bio2: A.b., and B. c. infection

In total, we identified 3492 distinct small RNAs from ASRP, MPSS, DB and the stress-treated dataset, which together match 914 of 2004 protein-coding cis-NAT genes (45.6%). This value is slightly higher than the proportion of non-NAT protein-coding genes with small RNA matches (42.6%). The difference in hit frequency is significant (p-value = 0.00436 estimated by bootstrap sampling as described in Materials and Methods).

The 914 cis-NAT genes with small RNA hits comprise 646 cis-NAT pairs (64.1% of the total protein-coding cis-NAT pairs) in *Arabidopsis *(Tables 5 and Additional file 4). This number is comparable with the probability that for two randomly paired non-NAT protein-coding genes, at least one will have a matching small RNA (63.5%).

The analysis of the subset of NATs with negatively correlated expression patterns (Pearson correlation coefficient <-0.4) showed increased frequency of small RNA hits (Table 7). Compared to NAT pairs with Pearson correlation score ≥ -0.4, the former has a higher frequency of small RNA hits both for the overlapping region (21.1% vs. 12.4%, p-value = 0.03499) and for full-length NATs (71.3% vs. 62.2%, p-value = 0.04992). Compared to the small RNA hit frequency for random pairs formed from non-NAT protein-coding genes, the full-length NAT hit frequency of the subgroup is higher (71.3% vs. 63.5%) but not statistically significant (p-value = 0.06612).

**Table 7:** Enrichment analysis of small RNAs in the negatively co-regulated NAT pairs

	NAT pairs	OL Hits	FL Hits
Pearson correlation < -0.4	71	15 (21.1%)	51 (71.3%)
Pearson correlation ≥ -0.4	595	74 (12.4%)	370 (62.2%)
Total	666	89 (13.3%)	421 (63.2%)

The p-value for the enrichment in the number of overlap regions (OL Hits) was 0.03499, and for the enrichment in the number of hits in the full-length NAT region (FL Hits) was 0.04992.

### Some putative nat-siRNAs may regulate more than one antisense gene

**Figure 3:**
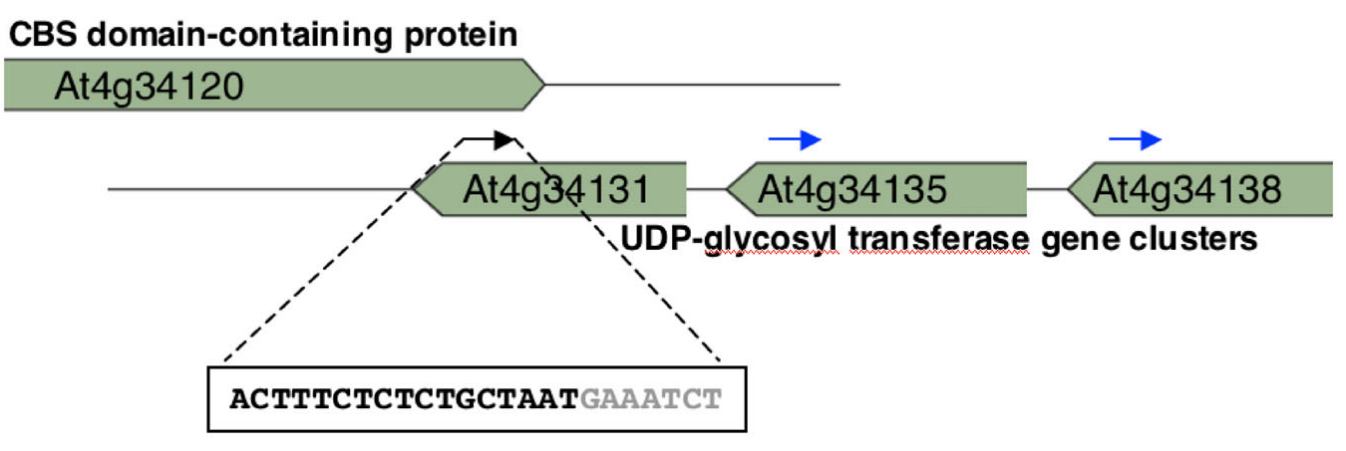
An example of one putative nat-siRNA (black arrow, sequence is indicated in the box) that can potentially silence all three UDP-glycosyl transferase genes: At4g34131, At4g34135 and At4g34138. Blue arrows indicate two more target sites.

In some cases, the siRNAs generated from cis-NATs match not only the genes in *cis *but also other mRNAs transcribed from non-overlapping loci in *trans*. Figure 3 shows an example of a small RNA with complementarity to three different transcripts, which thus may potentially target all of them. These three transcripts are highly similar to each other and all encode UDP-glycosyl transferases. An siRNA signature identified from the MPSS small RNA database matched the overlapping region of a CBS domain-containing protein gene (At4g34120) and one of the UDP-glucosyl transferase genes (At4g34131) and has the potential to silence all three of the UDP-glycosyl transferase genes, since all three of the transcripts have a 100% complementary match to the siRNA.

Interestingly, two ta-siRNA primary transcripts also overlap with other genes. TAS1C overlaps with a radical SAM domain-containing protein gene and generates many siRNAs (from MPSS, ASRP, DB and our stress-treated dataset) from both the sense and antisense strands of the locus, with some hitting the overlapping region. TAS2 also overlaps with a gene with protein-coding potential. Many siRNAs (from MPSS, ASRP, DB and the stress-treated dataset), including several ta-siRNAs that regulate pentatricopeptide-repeats protein genes in *trans*, are generated from both the sense and antisense strands of this locus.

Among the putative nat-siRNAs identified, 209 have complementarity with and may target more than one gene. We identified 103 putative nat-siRNAs that hit two or more genes within a gene family (Additional file 6). Consistent with our results, a recent study of Arabidopsis *trans*-NATs identified more than 400 NAT genes with both *cis *and *trans *partners [46]. Therefore, cis-NAT-generated siRNAs may play a broader role in gene regulation beyond that of the overlapping genes. They may regulate other gene family members or a group of genes with conserved small RNA target sites in trans and thus may be involved in complex gene regulatory networks.

### Functional annotation of Arabidopsis cis-NATs

**Figure 4:**
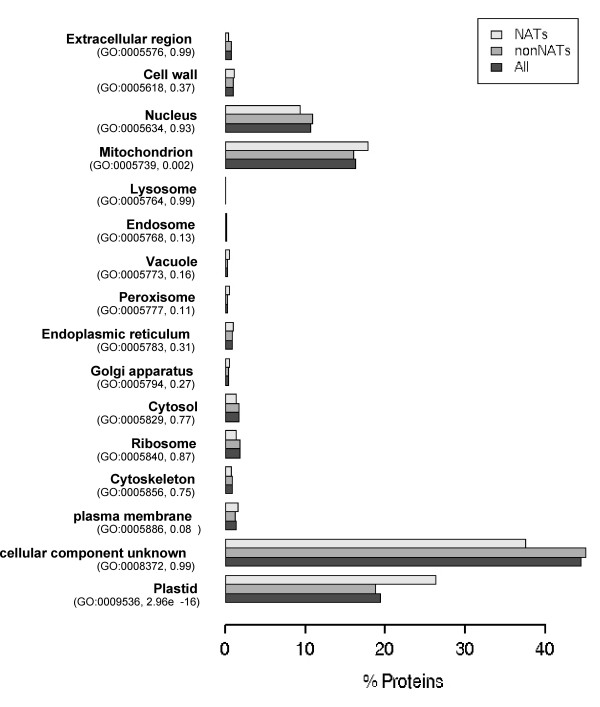
**Cellular component GO analysis**. The bar plot shows the relative distribution of cellular component GO slim categories among cis-NAT genes, non-NAT genes and all genes in the Arabidopsis genome. The p-values from the hypergeometric distribution test for the NAT genes are in parentheses after the corresponding GO identifiers.

We used enrichment analysis of Gene Ontology (GO) terms to functionally analyze the identified cis-NATs [47]. The entire cis-NAT set and the 593 pairs with inverse expression changes underwent statistical testing based on the hypergeometric distribution. We aimed to identify overrepresented terms by assigning a p-value to every node in the GO network [47]. In both sample sets the most highly enriched GO terms, among a total of 46 GO slim categories, were "catalytic activity" in the molecular function ontology with 641 and 310 sample matches at a node with 7591 associated genes (p-values ≤ 6.27e-06), and for the cellular component ontology it was "plastid" (420 and 210 sample matches at a node with 3852 genes, p-values ≤ 9.86E-15) and "mitochondria" (285 and 127 sample matches at a node with 3232 genes, p-value = 0.002). Figure 4 shows the relative distribution of cellular-component GO categories among cis-NAT genes, non-NAT genes and all genes in the *Arabidopsis *genome. Interestingly, 420 NAT genes (20%, p-value = 2.96e-16) are associated with plastids and 285 with mitochondria (13%, p-value = 0.0026). These results indicate that 33% of the NAT genes are involved in processes associated with organelles having presumed prokaryotic origins. Computational predictions of subcellular targeting by the TargetP program [48] generated similar results (data not shown).

To identify gene pairs in which the sense and antisense partners have related functions and locations, we searched all nodes in the three ontologies for common terms between the two members of each NAT pair. When considering only relatively specific GO terms with 3 or more ancestor nodes from the root of each ontology, we found 206 NAT pairs sharing at least one common GO term. Randomly selected pairs show a significantly lower number of common GO terms (p-value < 0.01). The detailed GO analysis of gene pairs is available in Additional file 7.

## Discussion

Eukaryotic genomes feature large intergenic spaces, but contain a significant number of overlapping genes that encode cis-NATs. The specific overlapping arrangement is presumed to have physiological benefits. However, the general mechanisms of cis-NAT regulation are still unknown. Our recent studies provided two examples of nat-siRNAs with an important role in regulating cis-NAT expression in response to abiotic and biotic stress [28,29]. Here, we performed a genome-wide analysis of Arabidopsis cis-NATs and discovered a group of NAT pairs with an inverse expression pattern between the sense and antisense transcripts in response to various stress treatments and/or at different developmental stages. This negative-expression correlation within a NAT pair, together with the finding that more than 200 NAT pairs share a common GO term, suggests that the overlapping arrangement is important for expression regulation of at least a subgroup of NAT genes in certain cellular processes or in response to stresses. We hypothesized that the induced expression of one transcript may pair with the existing antisense transcript within the same cell to form dsRNA, which can be processed into nat-siRNA(s). These nat-siRNAs can cause silencing of the antisense transcript. Although the negative-expression correlation within a NAT pair could also be caused by transcriptional interference, the identification of about 3500 putative nat-siRNAs from our stress-treated small RNA dataset and current MPSS, ASRP and DB small RNA databases, together with the observation that small RNAs are enriched in the overlapping regions of negatively correlated NAT pairs compared with non-negatively correlated pairs, strongly suggests that siRNA-mediated gene silencing may be one of the major molecular mechanisms of gene expression regulation of at least a subgroup of cis-NATs. Current small RNA cloning strategies may have not been able to identify nat-siRNAs present at very low levels or induced only transiently, by treatments of short durations or by a narrow window of treatment levels. This suggestion is also supported by little overlap of identified nat-siRNA candidates from different datasets. We expect to identify more nat-siRNAs by deeper coverage sequencing of small RNAs from plants treated with more stress conditions and more defined time intervals.

dsRNAs are formed during plant virus replication and transgene expression by viral RNA-dependent RNA polymerase (RDRs) or plant endogenous RDRs [30,49]. These dsRNAs are processed by Dicer-like proteins and generate siRNAs from both strands and cover the whole double-stranded region to induce silencing. Although the pairing of sense and antisense NAT transcripts also forms dsRNAs and triggers siRNA formation, our published work on three pairs of NATs suggests that nat-siRNAs are often generated from one specific strand and/or from one specific site to induce silencing of only one strand of the NAT pair [28,29,50]. To determine whether this is a general observation, we examined all the overlapping regions that have putative nat-siRNA hits, and revealed that 83% of these regions are targeted by strand-specific small RNAs. About 17% of these regions have small RNA hits on both strands, which can potentially silencing both the sense and antisense transcripts simultaneously. These results indicate that majority of the nat-siRNAs regulate only one transcript within a NAT pair and suggest that the formation of nat-siRNAs may require additional sequence and/or structural features of the overlapping transcripts or additional factors. This suggestion is supported by our recent study of a bacteria inducible nat-siRNA, nat-siRNAATGB2, whose induction by the bacterial pathogen *P.s.t*. carrying the avirulence gene *avrRpt2 *required not only the induction of the sense gene *ATGB2 *but also the presence of cognate resistance gene *RPS2 *and its resistance signaling component *NDR1 *[28]. The biogenesis of this nat-siRNA is also under the control of Dicer-like 1, RDR6, RNA polymerase IVa, HYL1 and HEN1 [28]. This finding indicates that multiple layers of control exist in nat-siRNA biogenesis and that the induction of the sense transcript of a NAT pair is necessary but may not be sufficient to induce some of the nat-siRNAs.

Our recent studies of stress-induced nat-siRNAs demonstrated that the biogenesis of these nat-siRNAs also depends on RDR6 and PolIVa subunit NRPD1a, the components required for RNA replication [28,29]. Thus, dsRNA formed by sense-antisense pairing might involve a secondary RNA replication loop to form nat-siRNAs. This RNA amplification step may extend beyond the overlapping region to form siRNAs outside the overlapping region. We hypothesize that these nat-siRNAs may ensure that the antisense gene is silenced.

Furthermore, "tiling" microarray studies to assay transcript levels throughout the genomes have revealed that more than 30% of the Arabidopsis genome and about 60–80% of mammalian genomes can produce transcripts from both strands [10,51]. About 7600 genes in Arabidopsis showed significant antisense RNA expression [46]. Interestingly, a recent study of rice involving robust-long serial analysis of gene expression revealed that many antisense transcripts were induced after the infection of a fungal pathogen *Magnaporthe grisea*, which induces rice blast disease [52]. Therefore, antisense transcription is not only limited to the NATs but also applies to many non-NAT genes. Antisense transcription potentially forms dsRNAs, which can be recognized by RNAi machinery and generate small RNAs to guide gene expression regulation. That antisense transcription can be induced by pathogen infection is of particular interest. We have discovered that many small RNAs are also induced by pathogen infection ([28] and unpublished data). We are currently exploring whether some of these pathogen-induced small RNAs may be generated by the induction of antisense transcription outside the NAT regions.

## Conclusion

We have performed a comprehensive gene expression analysis of Arabidopsis *cis*-NATs and discovered that a significant fraction of the sense and antisense partners of cis-NATs are anti-correlated, with inverse changes in expression in response to environmental or developmental cues. We identified nearly 3500 unique siRNAs that match cis-NAT genes. The siRNAs are enriched in the overlapping regions of negatively correlated NAT pairs, which supports our hypothesis that siRNA-mediated gene silencing may be one of the important mechanisms in gene regulation of some NAT genes.

## Methods

### Microarray analysis

Statistical analysis of microarray data involved the R environment for statistical computing [53], R packages from BioConductor [53,54] and custom R scripts.

#### Data sources

Raw expression data of 1310 Affymetrix ATH1 genome arrays were downloaded in Cel format from the public microarray repositories AtGenExpress [36] and Gene Expression Omnibus [37]. The chosen data sets are derived from 41 different experiment series covering a wide variety of treatment categories. In addition, the selected experiments contained at least two replicates, a requirement for statistical determination of differential gene expression. A detailed list of the downloaded data sets is in Additional file 1.

#### Sample Comparisons

To identify differentially expressed genes, we used a conservative approach to minimize the number of experimental factors within each experiment series. For instance, if an experiment series contained a specific treatment factor (*e.g*. cold) that was applied to different tissues at different time points, we performed only comparisons (contrasts) that tested the influence of the main treatment (*e.g*. cold) on global gene expression by comparing the same tissues at identical time points. Multi-factorial analyses were avoided as much as possible for the given experimental designs. To minimize the risk of including unknown variables into the analysis (*e.g*. inconsistent growth conditions between laboratories), comparisons between different experiment series were not considered. To automate the downstream analysis, information about replicated arrays and the chosen comparisons was organized in an experiment definition table (Additional file 1) that provided the required input parameters for the downstream analysis scripts.

#### Raw data analysis

Normalized and background-corrected expression values were extracted from probe-level Cel files by use of the Affymetrix's MAS 5.0 algorithm in the affy package from BioConductor [55].

#### Differential expression analysis

Differentially expressed genes were identified by use of a combination of fold-change analysis and Linear Models for Microarray Data (Limma) as a statistical test [38]. Genes were counted as differentially expressed if their expression values fulfilled the following criteria: (1) at least a 2-fold change and (2) an fdr-adjusted p-value < 0.01 in the Limma analysis. On the basis of complexity considerations, the experimental sets ME00319 and ME00331 (see additional file 1) were used only in the correlation analysis, not in the differential expression analysis.

Inverse expression changes of overlapping gene pairs were counted if one member in a pair was up-regulated and the other down-regulated according to the specified analysis criteria. In a more stringent analysis, these opposing expression changes had to occur in the same comparison, whereas a more loose analysis allowed them to be anywhere within a treatment series.

#### Correlation analysis

Negatively co-regulated gene pair members were also identified by comparing their expression profiles across all treatments. For this purpose the Pearson and Spearman correlation coefficients were calculated between members of each gene pair using a total of 560 centralized expression values derived from the averaged intensity values between replicates of the total dataset. The usage of two different types of correlation methods allowed us to identify false-positive correlations due to extreme expression changes (outliers) in certain samples.

#### Functional gene analysis

Gene Ontology analysis was performed with the GOstats package from BioConductor [47] and custom R scripts. The required probe sets to gene mappings were retrieved from the TAIR site, and the *Arabidopsis *GO annotations were downloaded from the Gene Ontology database [56,57].

### Sequence datasets

Arabidopsis gene sequences and annotations were downloaded from TAIR 6.0 release, September 2006 [58]. We based the analysis on the datasets for AGI Genes (gene sequences including introns and UTRs) and AGI Transcripts (containing exons and UTRs). Because this study focused on identifying siRNA candidates, we concentrated solely on the 28,194 protein-coding genes, obtained after removing 827 r,t,mi,sn,sno,snmRNA genes and 2,346 transposons and retrotransposons. Of these protein-coding genes, 2004 form 1008 NATs (12 have two overlapping regions, see Figure 1). In addition to this dataset, for small RNA discovery, we used coding regions with UTRs and whole-chromosome genomic sequences.

Small RNA libraries constructed from Arabidopsis plants treated with abiotic stress (cold, drought, salt, copper, UV and ABA treatments) and biotic stress (*P.s.t. DC3000 and DC3000 (avrRpt2)*, fungi *A. brassicicola *and *B. cinerea *infection) (Jin, *et al*., unpublished) were sequenced by 454 Life Sciences (Bradford, CT, USA) with pyrosequencing [44]. Adaptor sequences enclosing the inserts were trimmed, and the inserts were normalized to the sense direction. We discarded all inserts shorter than 18 bp. The remaining sequences were scanned for duplicates, and a non-redundant set was created.

Small RNA Arabidopsis MPSS [33] 17-bp signatures were downloaded from the University of Delaware . The Arabidopsis ASRP dataset [40] was downloaded from the Center for Genome Research and Biocomputing at Oregon State University . Sequences from the study by Rajagopalan *et al*. [35] were downloaded from NCBI [37], as Platform GPL3968; samples GSM118372, GSM118373, GSM118374 and GSM118375; and series GSE5228.

### Sequence discovery

Small RNA from the stress libraries, ASRP, MPSS and Rajagopalan *et al*. were aligned by use of BLAST [59] against the AGI Genes and AGI Transcripts datasets. BLAST queries were optimized for finding short, near-identical matches (seed word size 7, expected value 1,000). Small RNA from all four sets that matched coding regions, introns or UTRs of known t/r/mi/si/sno/sn/snmRNA, transposons or retrotransposons, as annotated in TAIR, were removed from the list of matches to provide a candidate dataset of small RNAs that would match protein-coding genes.

### Statistical significance of blast hit enrichment

We denote two sets of nucleotide sequences as *P *and *Q*, where the fraction of sequences from *P *with small RNA BLAST matches is higher than the corresponding fraction of sequences from *Q*: |*P*hits|/|*P*| > |*Q*hits|/|*Q*|. We use bootstrapping [60] to estimate the statistical significance of the hypothesis that small RNAs more frequently match *P *than *Q*. The null hypothesis is that a small RNA is equally likely to have a BLAST hit against *P *as against *Q*, and the test statistic used to estimate the p-value is that the relative frequency of a small RNA matching *P *was higher than that of a small RNA matching *Q*. Reported p-values are the number of occurrences of the event $\left|\hat{P}\;\text{hits}\right|/\;\left|\hat{P}\right|\leq \left|\hat{Q}\;\text{hits}\right|/\;\;\left|\hat{Q}\right|$ occurring by chance out of 100,000 bootstrap iterations, where in each iteration pseudo-replicate datasets of sequences, $\hat{P}$ and $\hat{Q}$, were created by sampling from *P *and *Q *with replacement.

### Genome cluster

The latest 70% BLASTCUST set from the Genome Cluster Database was used for sequence family analyses. In this data set the protein sequences were clustered with the BLASTCLUST program from NCBI using 50% overlap and 70% identity as cutoff values for family assembly [61].

## List of abbreviations

ds RNA: Double-stranded RNA; siRNA: Small interfering RNA; NATs: Nature antisense transcripts; RDRs: RNA-dependent RNA polymerase; TAIR: The Arabidopsis Information Resource; MPSS: Massively Parallel Signature Sequencing; ASRP: *Arabidopsis *Small RNA Project; ta-siRNA: Trans-acting siRNA; bp: Base pair.

## Authors' contributions

HJ and J-KZ designed the research; TG conducted the expression correlation and GO analyses of NATs; VV, HJ and SL conducted the small RNA analysis; HJ, TG and VV analyzed the data and wrote the paper; SL and J-KZ edited the paper. All authors read and approved the final manuscript.

## Supplementary Material

Additional file 1 All cis-NATs in Arabidopsis. Click here for file

Additional file 2 Summary of AtGenExpress experimental dataset. Click here for file

Additional file 3 NATs with inverse expression patterns. Click here for file

Additional file 4 Small RNAs with 100% sequence match to the overlapping regions of cis-NATs. Click here for file

Additional file 5 Small RNAs with 100% sequence match to the full-length cis-NATs. Click here for file

Additional file 6 List of putative nat-siRNAs that have the potential to target multiple members in a gene family. Click here for file

Additional file 7 Full dataset of the detailed GO analysis of NAT gene pairs. Click here for file

## References

[B1] Werner A, Berdal A (2005). Natural antisense transcripts: sound or silence?. Physiol Genomics.

[B2] Makalowska I, Lin CF, Makalowski W (2005). Overlapping genes in vertebrate genomes. Comput Biol Chem.

[B3] Boi S, Solda G, Tenchini ML (2004). Shedding light on the dark side of the genome: Overlapping genes in higher eukaryotes. Current Genomics.

[B4] Lavorgna G, Dahary D, Lehner B, Sorek R, Sanderson CM, Casari G (2004). In search of antisense. Trends Biochem Sci.

[B5] Zhang Y, Liu XS, Liu QR, Wei L (2006). Genome-wide in silico identification and analysis of cis natural antisense transcripts (cis-NATs) in ten species. Nucleic Acids Res.

[B6] Chen JJ, Sun M, Kent WJ, Huang XQ, Xie HQ, Wang WQ, Zhou GL, Shi RZ, Rowley JD (2004). Over 20% of human transcripts might form sense-antisense pairs. Nucleic Acids Research.

[B7] Yelin R, Dahary D, Sorek R, Levanon EY, Goldstein O, Shoshan A, Diber A, Biton S, Tamir Y, Khosravi RR, Nemzer S, Pinner E, Walach S, Bernstein J, Savitsky K, Rotman G (2003). Widespread occurrence of antisense transcription in the human genome. Nature Biotechnology.

[B8] Kiyosawa H, Yamanaka I, Osato N, Kondo S, Hayashizaki Y (2003). Antisense transcripts with FANTOM2 clone set and their implications for gene regulation. Genome Res.

[B9] Okazaki Y, Furuno M, Kasukawa T, Adachi J, Bono H, Kondo S, Nikaido I, Osato N, Saito R, Suzuki H (2002). Analysis of the mouse transcriptome based on functional annotation of 60,770 full-length cDNAs. Nature.

[B10] Katayama S, Tomaru Y, Kasukawa T, Waki K, Nakanishi M, Nakamura M, Nishida H, Yap CC, Suzuki M, Kawai J, Yamanaka I, Kiyosawa H, Yagi K, Tomaru Y, Hasegawa Y, Nogami A, Schonbach C, Gojobori T, Baldarelli R, Hill DP, Bult C, Hume DA, Quackenbush J, Schriml LM, Kanapin A, Matsuda H, Batalov S, Beisel KW, Blake JA, Bradt D (2005). Antisense transcription in the mammalian transcriptome. Science.

[B11] Misra S, Crosby MA, Mungall CJ, Matthews BB, Campbell KS, Hradecky P, Huang Y, Kaminker JS, Millburn GH, Prochnik SE, Smith CD, Tupy JL, Whitfied EJ, Bayraktaroglu L, Berman BP, Bettencourt BR, Celniker SE, de Grey AD, Drysdale RA, Harris NL, Richter J, Russo S, Schroeder AJ, Shu SQ, Stapleton M, Yamada C, Ashburner M, Gelbart WM, Rubin GM, Lewis SE (2002). Annotation of the Drosophila melanogaster euchromatic genome: a systematic review. Genome Biol.

[B12] Jen CH, Michalopoulos I, Westhead DR, Meyer P (2005). Natural antisense transcripts with coding capacity in Arabidopsis may have a regulatory role that is not linked to double-stranded RNA degradation. Genome Biology.

[B13] Wang XJ, Gaasterland T, Chua NH (2005). Genome-wide prediction and identification of cis-natural antisense transcripts in Arabidopsis thaliana. Genome Biology.

[B14] Osato N, Yamada H, Satoh K, Ooka H, Yamamoto M, Suzuki K, Kawai J, Carninci P, Ohtomo Y, Murakami K, Matsubara K, Kikuchi S, Hayashizaki Y (2003). Antisense transcripts with rice full-length cDNAs. Genome Biol.

[B15] Galante PAF, Vidal DO, de Souza JE, Camargo AA, de Souza SJ (2007). Sense-antisense pairs in mammals: functional/evolutionary considerations. Geome Biology.

[B16] Tufarelli C, Stanley JA, Garrick D, Sharpe JA, Ayyub H, Wood WG, Higgs DR (2003). Transcription of antisense RNA leading to gene silencing and methylation as a novel cause of human genetic disease. Nat Genet.

[B17] Prescott EM, Proudfoot NJ (2002). Transcriptional collision between convergent genes in budding yeast. Proc Natl Acad Sci USA.

[B18] Hastings ML, Milcarek C, Martincic K, Peterson ML, Munroe SH (1997). Expression of the thyroid hormone receptor gene, erbAalpha, in B lymphocytes: alternative mRNA processing is independent of differentiation but correlates with antisense RNA levels. Nucleic Acids Res.

[B19] Lee JT, Davidow LS, Warshawsky D (1999). Tsix, a gene antisense to Xist at the X-inactivation centre. Nat Genet.

[B20] Shibata S, Lee JT (2003). Characterization and quantitation of differential Tsix transcripts: implications for Tsix function. Human Molecular Genetics.

[B21] Shibata S, Lee JT (2004). Tsix transcription-versus RNA-based mechanisms in Xist repression and epigenetic choice. Current Biology.

[B22] Sleutels F, Zwart R, Barlow DP (2002). The non-coding Air RNA is required for silencing autosomal imprinted genes. Nature.

[B23] Thakur N, Tiwari VK, Thomassin H, Pandey RR, Kanduri M, Gondor A, Grange T, Ohlsson R, Kanduri C (2004). An antisense RNA regulates the bidirectional silencing property of the Kcnq1 imprinting control region. Mol Cell Biol.

[B24] Lewis A, Mitsuya K, Umlauf D, Smith P, Dean W, Walter J, Higgins M, Feil R, Reik W (2004). Imprinting on distal chromosome 7 in the placenta involves repressive histone methylation independent of DNA methylation. Nat Genet.

[B25] Peters NT, Rohrbach JA, Zalewski BA, Byrkett CM, Vaughn JC (2003). RNA editing and regulation of Drosophila 4f-rnp expression by sas-10 antisense readthrough mRNA transcripts. Rna.

[B26] Kim DDY, Kim TTY, Walsh T, Kobayashi Y, Matise TC, Buyske S, Gabriel A (2004). Widespread RNA editing of embedded Alu elements in the human transcriptome. Genome Research.

[B27] Aravin AA, Naumova NM, Tulin AV, Vagin VV, Rozovsky YM, Gvozdev VA (2001). Double-stranded RNA-mediated silencing of genomic tandem repeats and transposable elements in the D. melanogaster germline. Curr Biol.

[B28] Katiyar-Agarwal S, Morgan R, Dahlbeck D, Borsani O, Villegas A, Zhu JK, Staskawicz BJ, Jin HL (2006). A pathogen-inducible endogenous siRNA in plant immunity. Proceedings of the National Academy of Sciences of the United States of America.

[B29] Borsani O, Zhu JH, Verslues PE, Sunkar R, Zhu JK (2005). Endogenous siRNAs derived from a pair of natural cis-antisense transcripts regulate salt tolerance in Arabidopsis. Cell.

[B30] Baulcombe D (2005). RNA silencing. Trends Biochem Sci.

[B31] Vazquez F (2006). Arabidopsis endogenous small RNAs: highways and byways. Trends in Plant Science.

[B32] Chen J, Sun M, Hurst L, Carmichael G, Rowley JD (2005). Genome-wide analysis of coordinate expression and evolution of human cis-encoded sense-antisense transcripts. Trends in Genetics.

[B33] Lu C, Tej SS, Luo S, Haudenschild CD, Meyers BC, Green PJ (2005). Elucidation of the small RNA component of the transcriptome. Science.

[B34] Kasschau KD, Fahlgren N, Chapman EJ, Sullivan CM, Cumbie JS, Givan SA, Carrington JC (2007). Genome-Wide Profiling and Analysis of Arabidopsis siRNAs. PLoS Biol.

[B35] Rajagopalan R, Vaucheret H, Trejo J, Bartel DP (2006). A diverse and evolutionarily fluid set of microRNAs in Arabidopsis thaliana. Genes & Development.

[B36] AtGenExpress. http://www.arabidopsis.org/info/expression/ATGenExpress.jsp.

[B37] GEO http://www.ncbi.nlm.nih.gov/geo.

[B38] Smyth GK, Gentleman R, Carey V, Dudoit S, Irizarry R, Huber W (2005). Limma: linear models for microarray data. 'Bioinformatics and Computational Biology Solutions using R and Bioconductor'.

[B39] Henz SR, Cumbie JS, Kasschau KD, Lohmann JU, Carrington JC, Weigel D, Schmid M (2007). Distinct expression patterns of natural antisense transcripts in Arabidopsis. Plant Physiol.

[B40] Gustafson AM, Allen E, Givan S, Smith D, Carrington JC, Kasschau KD (2005). ASRP: the Arabidopsis Small RNA Project Database. Nucleic Acids Research.

[B41] Fahlgren N, Howell MD, Kasschau KD, Chapman EJ, Sullivan CM, Cumbie JS, Givan SA, Law TF, Grant SR, Dangl JL, Carrington JC (2007). High-Throughput Sequencing of Arabidopsis microRNAs: Evidence for Frequent Birth and Death of MIRNA Genes. PLoS ONE.

[B42] Lu C, Kulkarni K, Souret F, Muthuvalliappan R, Tej S, Poethig R, Henderson I, Jacobsen S, Wang W, Green P, Meyers B (2006). MicroRNAs and other small RNAs enriched in the Arabidopsis RNA-dependent RNA polymerase-2 mutant. Genome Res.

[B43] Llave C, Kasschau KD, Rector MA, Carrington JC (2002). Endogenous and silencing-associated small RNAs in plants. Plant Cell.

[B44] Margulies M, Egholm M, Altman WE, Attiya S, Bader JS, Bemben LA, Berka J, Braverman MS, Chen YJ, Chen Z, Dewell SB, Du L, Fierro JM, Gomes XV, Godwin BC, He W, Helgesen S, Ho CH, Irzyk GP, Jando SC, Alenquer MLI, Jarvie TP, Jirage KB, Kim JB, Knight JR, Lanza JR, Leamon JH, Lefkowitz SM, Lei M, Li J, Lohman KL, Lu H, Makhijani VB, McDade KE, McKenna MP, Myers EW, Nickerson E, Nobile JR, Plant R, Puc BP (2005). Genome sequencing in microfabricated high-density picolitre reactors. Nature.

[B45] 454. http://www.454.com.

[B46] Wang H, Chua N, Wang X (2006). Prediction of trans-antisense transcripts in Arabidopsis thaliana. Genome Biol.

[B47] Gentleman R, Jaromir Antoch (2004). Using {GO} for statistical analyses. Compstat 2004 Proceedings in Computational Statistics.

[B48] Emanuelsson O, Nielsen H, Brunak S, Heijne G (2000). Predicting subcellular localization of proteins based on their N-terminal amino acid sequence. J Mol Biol.

[B49] Voinnet O (2005). Induction and suppression of RNA silencing: Insights from viral infections. Nature Reviews Genetics.

[B50] Katiyar-Agarwal S, Gao S, Vivian-Smith A, Jin H (2007). A novel class of bacteria-induced small RNAs in Arabidopsis. Genes & Development.

[B51] Yamada K, Lim J, Dale J, Chen H (2003). Empirical analysis of transcriptional activity in the Arabidopsis genome. Science.

[B52] Gowda M, Venu RC, Li H, Jantasuriyarat C, Chen S, Bellizzi M, Pampanwar V, Kim H, Dean RA, Stahlberg E, Wing R, Soderlund C, Wang GL (2007). Magnaporthe grisea infection triggers RNA variation and antisense transcript expression in rice. Plant Physiology.

[B53] Team RDC (2006). R: A language and environment for statistical computing. R Foundation for Statistical Computing, Vienna, Austria. ISBN 3-900051-07-0. http://www.R-project.org.

[B54] Gentleman RC, Carey VJ, Bates DM, Bolstad B, Dettling M, Dudoit S, Ellis B, Gautier L, Ge Y, Gentry J, Hornik K, Hothorn T, Huber W, Lacus S, Irizarry R, Leisch F, Li C, Maechler M, Rossini AJ, Sawitzki G, Smith C, Smyth G, Tierney L, Yang JY, Zhang J (2004). Bioconductor: open software development for computational biology and bioinformatics. Genome Biol.

[B55] Irizarry RA, Gautier L, Bolstad BM, Miller Cwcf, Astrand M, Cope LM, Gentleman R, Gentry J, Halling C, Huber W, MacDonald J, Rubinstein BIP, Workman C, Zhang J (2006). affy: Methods for Affymetrix Oligonucleotide Arrays.

[B56] Berardini T, Mundodi S, Reiser L, Huala E, Garcia-Hernandez M, Zhang P, Mueller LA, Yoon J, Doyle A, Lander G, Moseyko N, Yoo D, Xu I, Zoeckler B, Montoya M, Miller N, Weems D, Rhee SY (2004). Functional annotation of the Arabidopsis genome using controlled vocabularies. Plant Physiol.

[B57] Consortium TGO (2000). Gene Ontology: tool for the unification of biology. Nature Genetics.

[B58] Rhee S, Beavis W, Berardini T, Chen G, Dixon D, Doyle A, Garcia-Hernandez M, Huala E, Lander G, Montoya M, Miller N, Mueller LA, Mundodi S, Reiser L, Tacklind J, Weems DC, Wu Y, Xu I, Yoo D, Yoon J, Zhang P (2003). The Arabidopsis Information Resource (TAIR): a model organism database providing a centralized, curated gateway to Arabidopsis biology, research materials and community. Nucleic Acids Res.

[B59] Altschul SF, Madden TL, Schaffer AA, Zhang JH, Zhang Z, Miller W, Lipman DJ (1997). Gapped BLAST and PSI-BLAST: a new generation of protein database search programs. Nucleic Acids Research.

[B60] Efron B, Tibshirani RJ (1993). An Introduction to the Bootstrap. Chapman and Hall, New York.

[B61] Horan K, Lauricha J, Bailey-Serres J, Raikhel N, Girke T (2005). Genome cluster database. A sequence family analysis platform for Arabidopsis and rice. Plant Physiol.

